# Emerging technologies for rapid phenotypic antimicrobial susceptibility testing of clinical isolates of bacteria

**DOI:** 10.1128/jcm.00674-25

**Published:** 2025-09-03

**Authors:** Jacob Rattin, Malcolm Boswell, Daniel Rhoads

**Affiliations:** 1 Department of Pathology and Laboratory Medicine, Cleveland Clinic 2569, Cleveland, Ohio, USA; 2 Independent MicroDx Consultancy Services, Tucson, Arizona, USA; 3 Department of Pathology, Cleveland Clinic Lerner College of Medicine of Case Western Reserve University 161821, Cleveland, Ohio, USA; 4 Infection Biology Program, Lerner Research Institute, Cleveland Clinic 2569, Cleveland, Ohio, USA; Vanderbilt University Medical Center, Nashville, Tennessee, USA

**Keywords:** blood culture, rapid antibiotic susceptibility testing, antibiotic susceptibility testing

## Abstract

Providing timely and accurate antimicrobial susceptibility testing (AST) results is a crucial component of clinical microbiology practice. Commercial rapid AST (RAST) is an emerging and quickly expanding area. These phenotypic RAST systems use various novel methods to monitor bacterial growth and replication in order to shorten the duration of time required for testing. Implementation of RAST has the potential to expedite antimicrobial therapeutic optimization, which can improve patient care. This minireview describes the current state of commercial phenotypic RAST including tests designed to report antimicrobial susceptibilities directly from clinical specimens.

## INTRODUCTION

Providing accurate and timely antimicrobial susceptibility testing (AST) interpretation is a crucial role in the clinical microbiology laboratory, which can improve patient care. Observational studies have identified that inadequate empiric antimicrobials and delays in starting appropriate antimicrobial treatment are risk factors for mortality (1, 2). Although no consensus definition of “rapid” AST (RAST) exists, one proposed definition will be employed in this review, which defines RAST as testing that can be initiated and completed within a single 8 h work shift (3). RAST is in contrast to reference AST methods that require 18–24 h of incubation of bacteria with antimicrobials.

**Fig 1:**
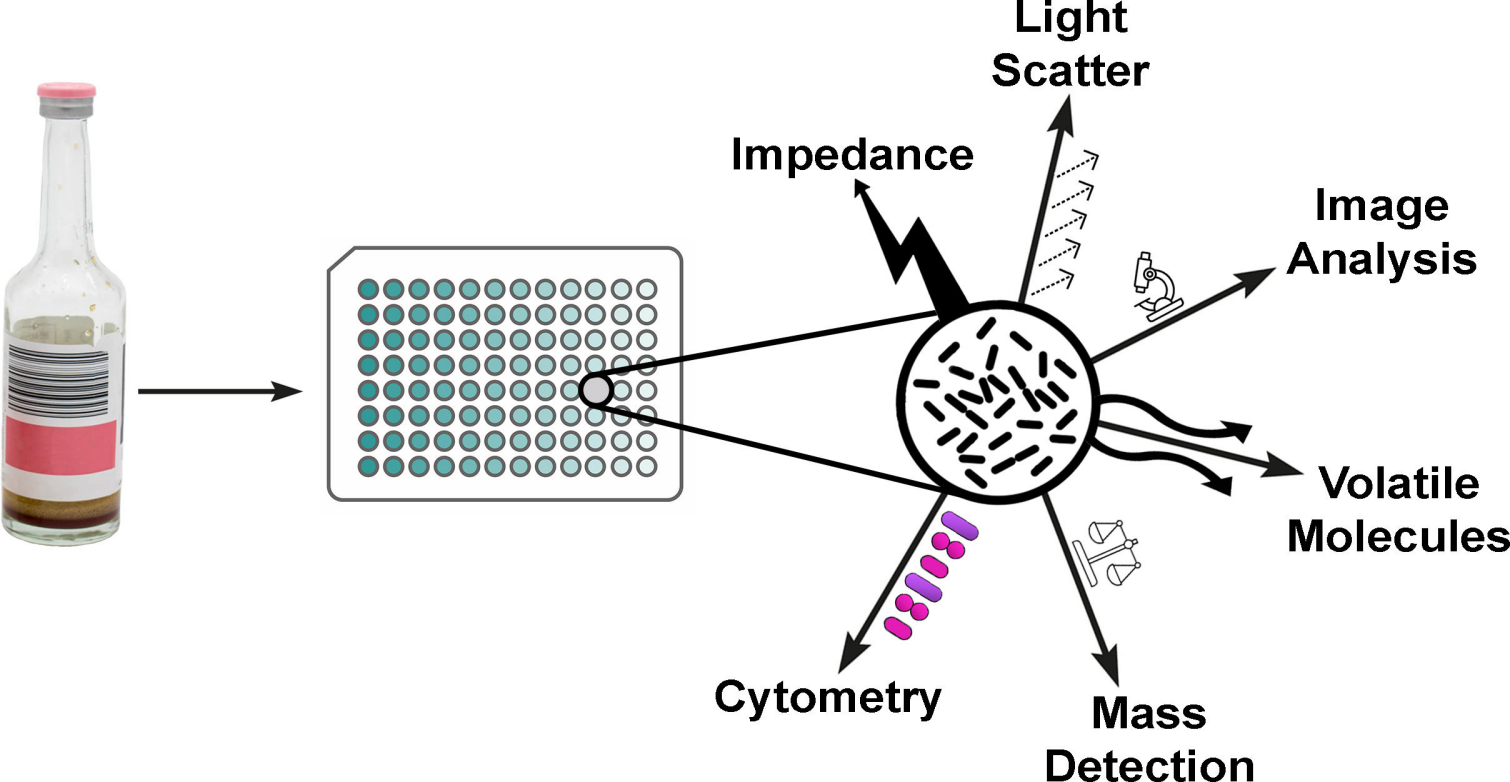
Generalized diagram of the workflow of various emerging technologies used for rapid phenotypic antimicrobial susceptibility testing. The first steps are the same wherein a positive blood culture or bacterial isolate (or, in some tests, a urine or respiratory sample) is aliquoted to a growth medium containing antimicrobials. The novelty of most emerging technologies is the method of assessment after incubation with the antimicrobials, which enables a shorter duration of incubation than standard methods. These emerging test systems use various analytical methods to measure the impact of antimicrobials on the bacteria, which can include electrical impedance, light scatter, microscopic image analysis, volatile molecule assessment, mass detection, or cytometry. These measured results are then assessed using the system’s novel criteria to determine the minimum inhibitory concentration and/or interpretation of susceptibility or resistance.

For more than half a century, clinical laboratories have been experimenting with ways to improve the rapidity of AST including modified commercial assays and fully manual methods (4, 5). This minireview will not review historical approaches for RAST, but will focus on contemporary and emerging phenotypic methods that have been commercialized or are in the commercialization pipeline. Phenotypic RAST methods include test systems that are designed to evaluate bacterial isolates by monitoring their growth, reproduction, metabolism, and/or inhibition thereof over time and in the presence or absence of defined concentrations of antimicrobial agents (3). Although not exclusively, many RAST systems are initially designed to test positive blood culture (PBC) broth because of the high mortality associated with inadequate therapy in cases of bacteremia and the accompanying high potential for positive impact that can occur with RAST in these cases. Numerous analysis methods can be used for performing phenotypic RAST on PBC (Fig. 1).

The inclusion of phenotypic RAST technologies and systems was based upon peer-reviewed literature, personal communication to companies via email, and public communications by the company or the FDA.

Unless otherwise defined by the respective study, herein time-to-result (TTR) is defined as the time from the start of the assay until the susceptibility results were reported by the instrument. Unless otherwise stated, the turnaround time (TAT) is the time from the start of the assay until the AST was reported.

## ON MARKET SYSTEMS: FDA-CLEARED AS OF 2025

### Accelerate diagnostics (Tucson, Arizona)

#### Accelerate Pheno system

The Accelerate Pheno system is the first technology available commercially in the United States to provide AST results directly from PBC and received initial FDA approval in 2017 (https://www.accessdata.fda.gov/cdrh_docs/reviews/DEN160032.pdf). A single aliquot of PBC is introduced to the test system, which both identifies the organism and determines the MIC for relevant antimicrobials.

Accelerate Pheno is unique, in that it performs identification in addition to AST, as other commercial systems used for AST direct from PBC require identification of the bacteria to be obtained using a separate test system. The Pheno’s identification results are generated via automated fluorescence *in situ* hybridization (FISH) analysis and are reported approximately 2 hours after starting the test (6). If the identified organism is a bacterial species that can be tested using the Pheno AST system, then MIC results are reported approximately 7 hours after starting the test (6).

The procedure is initiated by manual introduction of a 0.5 mL aliquot of PBC into the Pheno sample vial, which is performed in a biosafety cabinet. The sample vial, reagent cartridge, and 48-channel test cassettes are manually loaded into the Pheno equipment. Automated specimen preparation is performed via a process of gel electrofiltration, and distinct portions of the prepared sample are used for organism identification and the AST process.

Accelerate describes the AST methodology as morphokinetic cellular analysis (MCA), which takes place in the cassette channels. MCA is a computer vision-based analytical method that uses digital microscopy inputs from time-lapse dark field microscopy images of individual live cells and microcolonies. Machine learning algorithms track and analyze multiple bacterial morphological and kinetic changes, including cell morphology, cell mass as measured by light intensity of a growing microcolony, and division rate. Time-lapse imaging of bacterial cells in the presence and absence of antimicrobials is performed for up to 4.5 hours. Software algorithms determine MICs based on the MCA analysis, and corresponding categorical interpretations, SIR, are reported.

As Pheno has been commercially available longer than other contemporary RAST systems, more studies have evaluated the Pheno system than any other system discussed in this review. These studies often compare Pheno to the previous standard of care (SOC) methods to assess its performance in bloodstream infections with gram-negative bacilli (7), multidrug-resistant (MDR) gram-negative bacilli (8), both gram-positive and gram-negative organisms (9), and in a randomized trial to evaluate the test’s impact (10). The studies that included mean times to identification all reported a decrease in time to organism identification compared to SOC, ranging from an improvement of 23 h to 31 h (7, 9–11). Similarly, investigations that included time to AST results reported improved TTR using Pheno, with one study showing a nearly 42 h improvement (11).

Pheno has good overall identification performance, especially for its monomicrobial call (12–15). However, some highlighted discordant results in studies are failure to identify a case of *E. faecalis* and *S. aureus* (13), failure to detect organisms in polymicrobial infections (13, 15), and cross-reaction with α-hemolytic streptococci (14). Pheno has also shown overall accuracy with AST (12–16). However, some highlighted shortcomings include an AST failure rate of 8.2% and 8.6% in two studies (7, 15) and suboptimal performance of the antipseudomonal β-lactams for *P. aeruginosa* (12, 16). Finally, multiple instruments may be required since only a single sample can be run on an instrument. Please see Table 1 for more detailed data on performance.

**TABLE 1:** FDA-cleared test performance information^*a*^

Test	Overall CA	Overall EA	mE	ME	VME	Study
Accelerate PhenoTest BC Kit	GNB: 93.5% (2,159/2,306)	GNB: 93.7% (2,162/2,304)	GNB: 5.0% (116/2,306)	GNB: 1.3% (25/2,051)	GNB: 2.8% (6/220)	Freeman Weiss Z, Zelenkov D, Englert J, Campion M. Delving into discrepancies, a single-center experience with Accelerate Pheno for gram-negative bacteremia, a rapid phenotypic susceptibility testing method. Antimicrob Steward Healthc Epidemiol. 2025 Jan 23;5(1):e15. doi: 10.1017/ash.2024.482. PMID: 39911512; PMCID: PMC11795440 (17).
Accelerate PhenoTest BC Kit	For GPC: 97.9% (95% CI, 97.5 to 98.3%). For GNR: 94.3% (95% CI, 93.8 to 94.9%)	For GPC: 97.6% (95% CI, 97.1 to 98.1%). For GNR: 95.4% (95% CI, 94.9 to 95.9%)	For GPC: 1.3% (95% CI, 1.0 to 1.7%). For GNR: 4.8% (95% CI, 4.4 to 5.4%)	For GPC: 0.7% (95% CI, 0.4 to 1.0%). For GNR: 0.9% (95% CI, 0.7 to 1.3%)	For GPC: 1.0% (95% CI, 0.5 to 1.9%). For GNR: 0.5% (95% CI, 0.3 to 1.0%)	Pancholi P, Carroll KC, Buchan BW, Chan RC, Dhiman N, Ford B, Granato PA, Harrington AT, Hernandez DR, Humphries RM, Jindra MR, Ledeboer NA, Miller SA, Mochon AB, Morgan MA, Patel R, Schreckenberger PC, Stamper PD, Simner PJ, Tucci NE, Zimmerman C, Wolk DM. Multicenter Evaluation of the Accelerate PhenoTest BC Kit for Rapid Identification and Phenotypic Antimicrobial Susceptibility Testing Using Morphokinetic Cellular Analysis. J Clin Microbiol. 2018 Mar 26;56(4):e01329-17. doi: 10.1128/JCM.01329-17. PMID: 29305546; PMCID: PMC5869823 (12).
ASTar BC G-Kit	95.6% (832/870)	90.7% (754/831)	2.4% (21/870)	2.0% (12/593)	2.4% (5/212)	Esse J, Träger J, Valenza G, Bogdan C, Held J. 2023. Rapid phenotypic antimicrobial susceptibility testing of Gram-negative rods directly from positive blood cultures using the novel Q-linea ASTar system. J Clin Microbiol 61:e00549-23. https://doi.org/10.1128/jcm.00549-23 (18).
ASTar BC G-Kit	97.6% (8,433/8,639)	95.8% (8,283/8,650)	None Reported	0.9% (62/6,845)	2.4% (30/1,239)	Göransson J, Sundqvist M, Ghaderi E, Lisby JG, Molin Y, Eriksson E, Carlsson S, Cederlöf A, Ellis L, Melin J. Performance of a System for Rapid Phenotypic Antimicrobial Susceptibility Testing of Gram-Negative Bacteria Directly from Positive Blood Culture Bottles. J Clin Microbiol. 2023 Mar 23;61(3):e0152522. doi: 10.1128/jcm.01525-22. Epub 2023 Feb 28. Erratum in: J Clin Microbiol. 2024 Jan 17;62(1):e0117323. doi: 10.1128/jcm.01173-23. PMID: 36852983; PMCID: PMC10035315 (19).
Selux AST System	>90%	>90%	Not reported	GN: 0.7% (91/12,539) GP: 0.5% (16/3,067)	GN: 0.8% (24/2,934) GP: 0.8% (7/871)	Baker KR, Flentie K, Spears BR, Mozharov S, Roberts K, El ganbour A, Somers M, Calkwood J, Liu J, DaPonte K, Sam N, Kaur G, Chen F, Donato J, Chao A, Lewis A, Sherman J, Mortimer K, Harrington AT, Traczewski M, Carpenter D, Shortridge D, Lindley J, Diep A, Norton E, Green M, Gajewski J, Landrith R, Nalubega F, McCallum J, Beiswenger M, Dolan B, Brennan K, Carpenter A, Vacic A, Flyer AN, Pierce VM, Hooper DC, Lewis II JS, Stern E. 2024. Multicenter evaluation of the Selux Next-Generation Phenotyping antimicrobial susceptibility testing system. J Clin Microbiol 62:e00546-23. https://doi.org/10.1128/jcm.00546-23 (20).
LifeScale Gram Negative Kit	93.8% (N of tests = 633)	95.1% (N of tests = 642)	5.6% (N of tests = 37)	1.4% (N of tests = 8)	1.0% (N of tests = 1)	Montelongo-Jauregui D, Slechta ES, Akbari M, Fisher MA. LifeScale performance compared to Standard of Care (SOC) results. Affinity Biosensors (21).
LifeScale Gram Negative Kit	95.2% (1,031/1,081)	96.6% (1,046/1,082)	3.5% (38/1,081)	12 (only the number given) After resolution by BMD: 0.4% (4/947)	4 (only the number given) After resolution by BMD: 0.0% (0/120)	Snyder JW, Chaudhry N, Hoffmann W. 2024. Performance of the LifeScale automated rapid phenotypic antimicrobial susceptibility testing on Gram-negative rods directly from positive blood cultures. J Clin Microbiol 62:e00922-24. https://doi.org/10.1128/jcm.00922-24 (22)
Vitek Reveal GN AST Assay	96.3% (2,174/2,258) vs SensiTitre 96.2% (1,554/1,615) vs Vitek 2	98.0% (2,129/2,173) vs SensiTitre 97.0% (1,482/1,528) vs Vitek 2	3.5% (78/2,258) vs SensiTitre 3.3% (54/1,615) vs Vitek 2	0.3% (5/1,889) vs Sensititre 0.3% (4/1,342) vs Vitek 2	1.3% (4/313) vs SensiTitre 1.3% (3/232) vs Vitek 2	Tibbetts R, George S, Burwell R, Rajeev L, Rhodes PA, Singh P, Samuel L. Performance of the Reveal Rapid Antibiotic Susceptibility Testing System on Gram-Negative Blood Cultures at a Large Urban Hospital. J Clin Microbiol. 2022 Jun 15;60(6):e0009822. doi: 10.1128/jcm.00098-22. Epub 2022 May 24. PMID: 35607972; PMCID: PMC9199398 (23).
Vitek Reveal GN AST Assay	97.7% (808/827) simulated samples 99.2% (924/931) clinical samples	96.1% (816/849) simulated samples 98.8% (929/940) clinical samples	4.1% (12/295) simulated samples 1.0% (4/386) clinical samples	0.3% (1/312) simulated samples 0.3% (2/708) clinical samples	1.2% (6/491) simulated samples 0.4% (1/221) clinical samples	Menchinelli G, Squitieri D, Magrì C, De Maio F, D'Inzeo T, Cacaci M, De Angelis G, Sanguinetti M, Posteraro B. Verification of the Vitek Reveal System for Direct Antimicrobial Susceptibility Testing in Gram-Negative Positive Blood Cultures. Antibiotics (Basel). 2024 Nov 7;13(11):1058. doi: 10.3390/antibiotics13111058. PMID: 39596752; PMCID: PMC11590937 (24).

^*a*^ Studies highlighting FDA-cleared systems’ performances on categorical agreement (CA), essential agreement (EA), minor errors (mE), major errors (ME), and very major errors (VME). CI=confidence interval. Note: The test systems have variable amounts of available data published at the time of this writing.

A prospective, randomized clinical trial evaluated Pheno against SOC for gram-negative bacilli blood culture isolates. The aim was to compare the time to the first antimicrobial adjustment, use of antimicrobials, and patient outcomes (10). The time from PBC detection to the first antimicrobial modification was quicker when using Accelerate Pheno than SOC. The median (interquartile range, IQR) time to antimicrobial modification was 8.6 h (2.6–27.6 h) using Pheno vs 14.9 h (3.3–41.1 h) with the SOC (*P* = 0.02) (10). The frequency of change in gram-negative antimicrobials was more frequent in the Accelerate Pheno arm than in the SOC arm, and the time to gram-negative antimicrobial adjustment was shorter in the Pheno arm (17.3 h [4.9–72 h]) vs the SOC arm (42.1 h [10.1–72 h]) (*P* < 0.001) (10). In both arms, no difference in clinical outcomes, such as mortality, time to death, or length of hospitalization, was identified. For identification of bacteria that were on-panel, Pheno provided faster results than SOC, with a mean time of 2.7 h vs 14.5 h (*P* < 0.001). For AST results of bacteria on-panel, Pheno again provided faster results than SOC, 13.5 h vs 49.6 h (*P* < 0.001) (10).

Other studies examining time to targeted therapy show mixed results on inpatient mortality depending on the study design and patient population (10, 25, 26). No significant impact on mortality has been identified, but studies may not have been sufficiently powered or designed to detect a mortality impact. Consistent findings demonstrate GN antimicrobial modifications (antimicrobial escalation and de-escalation) occur more quickly than SOC (10, 25). Another study showed a reduction in time to optimal therapy when considering both gram-positive bacilli and gram-negative bacilli (26).

### Q-Linea AB (Uppsala, Sweden)

### ASTar instrument

Q-linea AB developed the ASTar instrument and gram-negative BC Kit and received FDA clearance in April of 2024 (https://www.accessdata.fda.gov/cdrh_docs/reviews/K221688.pdf). The TTR is approximately 6 hours as per the manufacturer (27). Organism identification is not provided by ASTar and must be obtained by an alternate SOC method.

The system performs broth microdilution (BMD) testing directly from PBC and provides MICs for up to 23 antimicrobials for non-fastidious gram-negative bacilli and six antimicrobials for fastidious gram-negative bacilli (19). To perform the test, a PBC is first identified as containing gram-negative bacilli by Gram stain. Then, a 1 mL aliquot is added to the ASTar BC G− cartridge, which is loaded onto the ASTar instrument along with a consumable kit containing a disk. Of note, the reagent insert needs to be stored frozen until use. The user needs to identify the bacterial species using the SOC technology and share the identification with the ASTar system before the instrument can interpret the AST results. This can be done at the start of the run, during, or after (19). The ASTar output is MICs with categorical interpretations of SIR.

Within the instrument, the bacteria are recovered from the PBC via filtration and introduced to cation-adjusted Mueller-Hinton broth (CAMHB) (19). An aliquot of the bacterial suspension is used to determine the bacterial load (cells/mL), and the suspension is diluted by the instrument to a target density that is suitable for the AST. Some of the suspension is diluted in CAMHB, and another portion is diluted into an enriched medium that aids in the growth of fastidious bacteria. MIC testing occurs in the disk, which contains over 330 culturing chambers with various antimicrobial concentrations spanning six to fourteen two-fold dilutions for each antimicrobial (27). Bacterial growth within the disk chambers is identified by time-lapse imaging, and proprietary algorithms translate the image data into MICs. Once the species identity is entered into the ASTar system, the MIC and corresponding SIR interpretation are reported based on interpretive criteria. Up to six PBC samples can be loaded simultaneously onto a single instrument in a random-access manner, and up to 12 disks can be incubating simultaneously in the system.

Before receiving FDA clearance, ASTar has been available for clinical use outside of the United States (ex-U.S.). A study aimed at assessing susceptibility testing for gram-negative isolates from PBCs of the ASTar performance compared to reference BMD (frozen SensiTitre plates) has been performed (19). The retrospective arm used a study isolate bank (*N* = 412 isolates from clinical laboratories) and the prospective arm included PBCs of monobacterial, gram-negative bacteria (*N* = 74) identified via MALDI-TOF (Biotyper; Bruker) (19). The ASTar system showed reliable accuracy with an overall essential agreement (EA) of 95.8% and a categorical agreement (CA) of 97.6%, but TTR and/or TAT was not evaluated (Table 1). A prospective study using PBC with monomicrobial gram-negative bacilli that examined TTR compared to disk diffusion AST found the time range between the PBC flagging positive to ASTar output to be 6–24 h. The disk diffusion AST results reported in a range of 21–42 h, a difference of 17 hours compared to ASTar (28). However, 23 out of 68 episodes had a categorical disagreement between ASTar and the disk diffusion AST outcome, with most discrepancies being with amoxicillin-clavulanic acid (10 major errors) (28). This study further evaluated 68 episodes regarding therapeutic decisions. In 76% (52/68) cases, the patient was already on empirical antimicrobial therapy when the Gram stain notification was provided, and in 21% of these cases, the therapy was changed (e.g., change of antimicrobial or change of dose). ASTar identified resistance for ineffective antimicrobial therapy being used and assisted in switching to effective antimicrobial therapy in 17.6% (12/68) with a median time to change of 1 h after result release and provided opportunities for optimizing antimicrobial therapy in 63% of cases (43/68) (28).

A separate evaluation of ASTar found it correctly identified patients who required an escalation of therapy but failed to identify 5 of 20 patients (25%) who could have had de-escalated therapy (18). ASTar would have led to unnecessary escalation of therapy in two (4.7%) patients. It correctly guided antibiotic therapy in 84.1% of cases. In comparison, the standard method resulted in correct recommendations in 44 out of 46 patients (95.7%) (18).

### Selux diagnostics (Charlestown, Massachusetts)

#### Selux AST system

Selux Diagnostics received FDA clearance for the Selux AST system for gram-positive isolates (January 2023), gram-negative isolates (April 2023), and PBC gram-negative bacteria (February 2024) (https://www.accessdata.fda.gov/cdrh_docs/reviews/K211748.pdf). Identification must be performed with an alternative SOC method and entered into the system for susceptibility results to be reported. The average TTR is 5.5 hours as per the manufacturer (29).

The company describes its AST methodology as Next-Generation Phenotyping (NGP). After adequate growth is identified in the antimicrobial-free control, then two probe-based assays are performed in wells containing doubling dilutions of antimicrobials to determine the MIC and provide AST interpretations.

The system has three main component instruments: Sample Preparation Workstation, Inoculator, and Analyzer. The Sample Preparation Workstation prepares the bacterial suspension from an isolate and is also used for reviewing results. The inoculator transfers the culture suspension into AST panels where the bacteria are incubated with antimicrobials. The analyzer monitors the bacterial growth in the panels and performs the fluorescent assessment to determine the AST results.

The AST Panels are 384-well microplates, enabling the testing of up to 40 antimicrobials on each isolate covering a wide range of doubling dilutions. Bacterial growth is monitored using a fluorescent indicator dye, and once sufficient growth is recognized, then endpoint fluorescent readings are obtained using a “Viability assay” and a “Surface Area assay.” These fluorescent measurements are then used to determine the MICs, and results can be viewed on the Workstation.

At the time of this writing, there are 22 cleared combinations for gram-negative isolates, 13 combinations for gram positives, and 17 combinations for gram negatives direct from PBC (30). Only the Gram type of the organism is needed before AST processing, but species identification is required for reporting AST results.

A multi-institutional study evaluated the performance of the Selux AST system using 15 gram-positive antimicrobials and 24 gram-negative antimicrobials. The essential agreement exceeded the FDA minimum requirements of >89.9% (with the exception of *P. aeruginosa* with ceftazidime-avibactam [89.6%]) (Table 1). The average TTR for all species of both the clinical and challenge culture isolates was 5.56 h (20).

At the time of this writing, there is no known published data on the impact of use (e.g., time to targeted therapy).

### Affinity biosensors (Santa Barbara, California)

#### LifeScale AST system

The LifeScale AST system and Gram Negative Kit were cleared by the FDA in April of 2024 (https://www.accessdata.fda.gov/cdrh_docs/reviews/K211815.pdf) and in October of 2024 received additional clearance for updated performance data and MIC breakpoints for some species/drug combinations (https://www.accessdata.fda.gov/cdrh_docs/reviews/K241324.pdf). Organism identification must be provided by an alternate SOC method. The LifeScale workflow TTR takes 4.5 hours as per the manufacturer (31).

To determine the antimicrobial susceptibility, a PBC sample is grown in a microtiter plate with CAMHB broth and antimicrobials in a twofold dilution series. Then, the bacteria are assessed by the instrument. The LifeScale technology uses a sensor that detects the resonance of a microcantilever as the bacterial cells pass by it. The resonance is used to calculate the mass of each of the bacteria. The number and mass of the cells are used to determine whether or not the bacteria were inhibited by the antimicrobials with which they were incubating (32). The LifeScale software (i.e., Artificial Intelligence Predictor) determines the MIC values and interpretive results (SIR).

LifeScale was assessed with 100 clinical samples of gram-negative bacilli compared to SOC. When time was measured from the PBC, Gram stain, Verigene identification, and LifeScale, the TTR was within 7.2 h for LifeScale compared to a range of 36–40 h for SOC (22). The median TTR on the LifeScale system was 4 h and 30 minutes (range of 4–5 h and 50 min) (22). LifeScale was compared to SOC for EA and CA, achieving over 90% for all antimicrobials tested, except for cefazolin and piperacillin/tazobactam (Table 1) (22).

At the time of this writing, there is no known published data on the impact of use (e.g., time to targeted therapy).

### bioMérieux (Marcy-l'Étoile, France)

#### Vitek Reveal AST system

Vitek Reveal was developed by Specific Diagnostics (San Jose, CA) and acquired by bioMérieux in 2022. The AST system was FDA-cleared in June of 2024 (https://www.accessdata.fda.gov/cdrh_docs/reviews/K230675.pdf) and provides TTR in an average of 5.5 h as per the manufacturer (33).

The system determines MICs from PBC for gram-negative bacilli using an array of proprietary colorimetric chemical sensors, deemed small molecule sensors, that detect volatile compounds emitted from metabolically active cells in broth culture in a microtiter plate. The sensor arrays are printed onto a porous panel and positioned above each well on the microtiter plate. The microtiter plate with its panel of arrays is sealed together and then placed in the Vitek Reveal instrument, which serves as both an incubator and optical monitor of the sensors. Monitoring of the sensors occurs every 10 minutes, enabling real-time evaluation of bacterial metabolism in each well. A real-time algorithm analyzes sensor changes, detecting volatile compound emissions linked to bacterial proliferation. Each well containing an antimicrobial agent is compared to control wells—one serving as a positive control (lacking antimicrobial agents) and the other as a negative control (lacking growth media). When the sensors detect bacterial metabolism, then antimicrobial resistance is inferred. The MIC is determined, and categorical interpretation (SIR) is provided. An extended-spectrum beta-lactamase (ESBL) screen is also included in the testing.

One study evaluated Reveal’s performance using clinical PBC samples and a contrived challenge set of samples containing bacteria from the Centers for Disease Control’s Antimicrobial Resistance Isolate Bank (23). The average TTR was 4.6 h (3.0–6.8 h) for all species/drug pairs in the clinical samples and 4.3 h for the spiked MDR isolates (23). Another study evaluated the Reveal to the ISO 20776-1 BMD method for both simulated and clinical PBC samples. The study found that the TTR for resistant isolates was approximately 1 hour shorter than the TTR for isolates that tested intermediate or susceptible (24). The performance data were comparable to other studies (Table 1).

At the time of this writing, there is no known published data on the impact of use (e.g., time to targeted therapy).

## ADDITIONAL AST TECHNOLOGIES

The following technologies are potentially “near United States (U.S.) market entry.” As of the time of writing, these technologies have not received FDA regulatory approval or clearance to be used as *in vitro* diagnostic (IVD) devices, but the commercial developers/manufacturers intend to bring these platforms to market as IVDs in the United States according to public statements from the company or personal communications of the companies with the authors. Some of these systems are already on the market ex-U.S.

Accelerate Diagnostics (Tucson, Arizona), which has the Pheno system on market, submitted clinical trial data on the Accelerate WAVE system and positive blood culture gram-negative test kit to the FDA for 510(k) clearance on 21 March 2025. Unlike the Pheno system, the WAVE does not provide organism identification; therefore, it relies on other identification methodologies such as molecular methods or MALDI-TOF. Although both Pheno and WAVE employ microscopic analysis to determine AST, Pheno uses dark field microscopy, and WAVE uses lens-free holographic microscopy (34). Each WAVE module incorporates laser diodes and digital sensors to create two-dimensional holograms that can be reconstructed into three-dimensional images to assess microbial morphologic changes in the presence and absence of varying antimicrobial concentrations. Algorithms are applied to generate MIC results, and they can provide an MIC by assessing the cellular morphological response to antimicrobials (34). The range of time to an MIC result in one study was 3.0–4.7 h, with an average time of 4 h (34). The average time to MIC quoted in the press release for FDA submission is 4.5 h. Each WAVE module is capable of testing up to 10 samples in parallel, and up to five WAVE modules can be linked together into a single system.

Astek (Baltimore, Maryland) created the JIDDU System, a benchtop analyzer for identifying a bacterial urinary tract infection (UTI) and performing AST (35). The urine sample is placed into a microfluidic cartridge, which is loaded onto the instrument. The sample is divided into channels, admixing it with antimicrobials. In the channels, the sample is cultured for 35 minutes, and then analysis is performed, which takes about 15 minutes. The device output confirms the presence of bacteria and AST results. As per the manufacturer, the TAT of the process is 1 h (35). The company reported completion of a JIDDU alpha feasibility study performed on 50 urine samples from neurogenic bladder syndrome patients with suspected UTIs and plans clinical trials for the JIDDU Beta prototype in 2025.

Avails Medical (Menlo Park, California), in October of 2024, announced the launch of a U.S. pilot trial for the eAST system for PBC susceptibility testing. The eAST methodology is based on the company’s electrical biosensor technology, consisting of two electrodes (reference and indicator) that measure changes in oxidation-reducing potential that occurs when bacteria metabolize (36). The company claims that, because changes in oxidation-reducing potential are proportional to the load of pathogens, eAST is able to quantify bacteria in PBC samples in the presence of antimicrobials. The Avails eAST has 96 integrated electronic sensors in a disposable lid that fits onto a 96-well AST plate and reportedly has a TAT within 4 h (37).

Bacteromic (Warsaw, Poland) has developed Bacteromic system 2.0 technology, based on traditional BMD methods, that uses 640 microfluidic testing wells to assess over 30 antimicrobials using numerous dilutions. The system is random access, and up to 10 samples at a time can be loaded into the instrument with 60 samples able to be on the instrument at one time. The company is developing their system for both traditional (16 h) and rapid (6–8 h) susceptibility testing of bacterial isolates and PBC.

FASTinov (Porto, Portugal) has developed a flow cytometry methodology AST technology for use on PBC. The test uses fluorescent probes and measures cell lesions or metabolic changes. Proprietary computation then interprets the flow cytometry information to provide TTR within 2 h. The test is currently CE-IVD-marked.

First Light Diagnostics/Sansure (Chelmsford, Massachusetts) is developing UTI-Direct that performs susceptibility testing directly on urine specimens. The technology uses nonspecific magnetic tagging of bacteria and fluorescent labeling of bacterial cells by FISH. UTI-Direct consists of a two-step testing process of identification and AST with both steps performed on the company’s MultiPath Analyzer. The company claims a 6 h TTR.

Gradientech (Uppsala, Sweden) has developed QuickMIC, an instrument with cassettes able to analyze one PBC per run to provide an MIC in 2–4 h (38). The system uses microfluidics and live images to assess microcolony growth with a semisolid medium containing a linear antimicrobial gradient. The software will analyze the growth patterns of each microcolony to provide an MIC and SIR interpretation. Each cassette contains 12 antimicrobials for a gram-negative panel. The average TTR in one study was 182 minutes (39). In October 2024, Gradientech announced that Hardy Diagnostics would be the exclusive distributor of QuickMIC in the United States and Canada (https://gradientech.se/cision-feed/gradientech-in-exclusive-partnership-with-hardy-diagnostics-for-the-us-and-canadian-markets/). The company reported starting its U.S. clinical trial in September 2024.

iFAST Diagnostics (Southampton, United Kingdom) has the iFAST system, which stands for impedance-based Fast Antimicrobial Susceptibility Test. The system measures changes in the morphological and electrical characteristics of thousands of single organisms via microfluidic impedance cytometry utilizing a multi-electrode, microfluidic impedance chip (40). The electrical field in the chip comprises selected frequencies that have been shown to be indicative of specific biophysical properties of bacteria such as cell volume and membrane/cell wall permeability. An inoculum from an overnight bacterial culture is taken and resuspended in a growth medium for 30 minutes and incubated for another 30 minutes in the presence of an antimicrobial. About 10^5^ bacteria are measured in 2–3 minutes, generating a response profile. The manufacturer states the TTR is less than 3–4 h from PBC or a raw urine sample (41). The company initiated their U.K. market clinical trial for PBC and isolates (both gram negatives and gram positives) in April 2025 and also plans future U.S. clinical trials.

Pattern Bioscience, Inc. (Austin, Texas) is developing the Digital Culture System, which provides simultaneous identification (ID) and AST directly from respiratory samples and PBC. The technology uses a proprietary single-cell analysis method to measure individual bacterial cells and measures the metabolic response in picoliter-scale droplets (42). The assay is performed within multiple individual incubation chambers, therefore separating the response to antimicrobials for different bacteria in the sample. AI and pattern recognition are used to determine identification and susceptibility results. The technology is designed to detect and report AST results for more than one bacterial species in polymicrobial samples, which is particularly challenging for many other RAST technologies. A pneumonia ID/AST panel for respiratory specimens (bronchoalveolar lavage and endotracheal aspirate) and a bacteremia ID/AST panel for positive blood culture specimens are in development. A recent poster from ESCMID Global 2025 included data from 166 lower respiratory tract specimens from four U.S. medical centers using 2024 U.S. FDA/STIC breakpoints (43). Reference methods were MALDI-TOF MS for microorganism identification and disk diffusion for AST. An on-panel pathogen was identified in 56 of the 166 tested specimens (34%), and there were two false-negative results for above-threshold pathogens identified by reference methods. AST CA was 98.8%, with no VME reported. Of note, six specimens had two or more microbes detected with 100% AST CA in these polymicrobial specimens.

PhAST Diagnostics (Boston, Massachusetts) has developed PhAST, a cloud-based platform that uses single-cell imaging (44). The company (personal communication with authors) reported starting their U.S. clinical trial in 2024, intended for FDA 510(k) submission.

QuantaMatrix (Seoul, South Korea) has made dRAST, which can provide AST directly from blood. It uses a microfluidic agarose channel system that immobilizes bacteria. The bacterial growth is then evaluated by time-lapse imaging in different antimicrobial cultures (45, 46). The overall average TTR in one study was 6 h with a range of 4.7–7.9 h (46).

Resistell (Muttenz, Switzerland) has developed an AST system based on nano motion measurements of a cantilever with bacteria attached by a micromechanical sensor. The company positions the test as phenotypic and independent of bacterial growth, which is proposed to decrease the TTR. The company is also developing Phenotech MultiStar, a fully automated high-throughput diagnostic device with a 2 h TTR and intends to enter clinical trials in 2026.

Sysmex (Norderstedt, Germany), PA-100 AST System, uses a nanofluidic chip with 11,000 nanochannels to perform RAST for the most common uropathogenic bacteria in uncomplicated UTIs (47). The sample flows through the chip, where single bacterial cells are captured in separate channels, while larger cellular constituents are filtered out. Bacteria are incubated in broth and simultaneously exposed to five different antimicrobials at various concentrations: amoxicillin/clavulanic acid, ciprofloxacin, fosfomycin, nitrofurantoin, and trimethoprim. Contrast-phase microscopy is used to observe bacterial growth and reproduction. The resistant bacteria maintain a higher growth rate during incubation, whereas susceptible bacteria grow slowly or undergo lysis. Software interprets the microscopic images and reports AST results. The system is designed to be used in a point-of-care setting with the TAT being within an hour (48).

Utilizer (Stockholm, Sweden) is developing the UTIlizer-AST, a miniaturized, phenotypic growth-based colorimetric assay in a dipstick format, capable of detecting bacterial growth in the presence of different antimicrobials and providing susceptibility results directly from urine specimens. The company is conducting feasibility studies with prototypes in collaboration with KTH Royal Institute of Technology, Department of Micro- and Nanosystems, and Karolinska Institute in Sweden. The Utilizer-AST is intended to be a near-patient test that will use a smartphone for digital results with a TAT within 6–8 h.

The following companies are not discussed in this review but are developing technologies for phenotypic RAST (Table 2).

**TABLE 2:** Companies and their RAST product under development

Company and location	Product name
Acenxion Biosystems (Kansas City, Kansas)	fAST system
Alifax (Alifax, Italy)	Alfred 60/AST System
ArcDia International Oy Ltd (Turku, Finland)	mariAST
BacterioScan (St. Louis, Missouri)	BacterioScan 216Dx
Cytecom (Coventry, United Kingdom)	CyteCount
Cytophenix (Perth, Australia)	FloCAST
CytoSPAR (Beaufort, South Carolina)	BLAST
FluretiQ (Bristol, United Kingdom)	SCFI
HelixBind (Boxborough, Massachusetts)	Name not yet available
InnotiveDx (Bath, United Kingdom)	Innotive UTI
Microplate Dx (Glasgow, Scotland)	RapidPlate
Momentum Bioscience (Witney, United Kingdom)	SepsiSTAT
Occhino (Angleur, Belgium)	Ipac 2 AR
Prompt Diagnostics LLC (Baltimore, Maryland)	Name not yet available
RID Inc (Calgary, Canada)	Hopper: UTIDx and BSIDx
Shanx Medtech (Eindhoven, Netherlands)	KAIROS
SpeeDx (Sydney, Australia)	InSignia
TGV Dx (New York, New York)	AbtFinder
TTP and ODx Innovation (Melbourn, United Kingdom)	Name not yet available
Zhuhai DL Biotech (Zhuhai City, China)	D2 MiniMicrobial ID/AST system

## CONCLUDING CONSIDERATIONS FOR NOW AND THE FUTURE

Many new and established companies are working to improve the rapidity with which AST can be performed for clinical testing. In the past couple of years, several commercial AST IVD systems have received FDA clearance for RAST (Table 3). Although achieving regulatory clearance is a substantial hurdle, implementing these systems in clinical practice has additional challenges. Simons and Capraro have recently written a thoughtful description of the barriers to successful and impactful implementation of these RAST systems, which can include financial costs, technical expertise, existing workflows, stakeholder buy-in, and the rapidity of post-analytical action (49).

**TABLE 3:** FDA-cleared test information^*a*^

Test	FDA approval date	Identification menu and method	Identification time to result	AST method	AST menu	AST results	AST time to result	Specimen type	Number of antimicrobials tested	Bacteria menu	website
Accelerate Phenotest BC Kit	February 2017	Gram-negative and gram-positive bacteria, *C albicans*, *C glabrata*; Fluorescence *in situ* hybridization	2 h	Morphokinetic cellular analysis via automated time-lapse darkfield microscopy imaging	Gram-negative and Gram-positive bacteria	Minimum inhibitory concentrations (MICs) for gram-negative and gram-positive bacteria and categorical interpretations (SIR)	7 h	Positive blood culture	18 (Box 1)	Gram negative and gram positive	https://acceleratediagnostics.com/
ASTar BC G-Kit	April 2024	No identification performed	N/A	High-speed, time-lapse microscopic imaging of cells in broth microdilution	Gram-negative bacteria	MICs and interpretive results (SIR)	6 h	Positive blood culture	18 (Box 2)	Gram negative	https://qlinea.com/
Selux AST System	January 2023, April 2023, and February 2024	No identification performed	N/A	Colorimetric, oxidation reduction, and growth-based detection using metabolic indicators to detect organism growth viability signals	Gram-negative and gram-positive bacteria	Bacterial isolates: MICs for gram-negative and gram-positive bacteria; PBC: MICs and interpretive results (SIR) for gram-negative bacteria	5.5 h	Positive blood culture and colony isolates	23 (Box 3)	Gram negative and gram positive	https://seluxdx.com/
LifeScale Gram Negative Kit	April 2024	No identification performed	N/A	Counts cells and determines cell mass using microfluidic sensors and resonant frequency	Gram-negative bacteria	MICs and interpretive results (SIR)	4.5 h	Positive blood culture	14 (Box 4)	Gram negative	https://affinitybio.com/
Vitek Reveal GN AST Assay	June 2024	No identification performed	N/A	Chemical sensors monitor volatile compound emissions from metabolizing bacteria	Gram-negative bacteria	MICs and interpretive results (SIR)	5.5 h	Positive blood culture	22 (Box 5)	Gram negative	https://www.biomerieux.com/corp/en.html

^*a*^ Comparative information on each FDA-cleared RAST. N/A = Not Applicable.

Box 1. **Antimicrobial Agents Tested by the Accelerate Pheno System**

- Amikacin
- Ampicillin
- Ampicillin/sulbactam
- Aztreonam
- Ceftazidime
- Ceftaroline
- Cefepime
- Ceftriaxone
- Ciprofloxacin
- Daptomycin
- Erythromycin
- Ertapenem
- Gentamicin
- Linezolid
- Meropenem
- Piperacillin/tazobactam
- Tobramycin
- Vancomycin

Resistance Phenotypes Reported by the Accelerate Pheno System

- Methicillin-resistance

Note: For the details on the FDA-cleared combinations of antimicrobial agents that can be tested with specific taxa, refer to the instructions for use.

Box 2. **Antimicrobial Agents Tested by the Q-Linea ASTar Instrument**

- Amikacin
- Ampicillin
- Ampicillin-Sulbactam
- Aztreonam
- Cefazolin
- Cefepime
- Ceftazidime
- Ceftazidime-Avibactam
- Cefuroxime
- Ciprofloxacin
- Gentamicin
- Levofloxacin
- Meropenem
- Meropenem-Vaborbactam
- Piperacillin-Tazobactam
- Tigecycline
- Tobramycin
- Trimethoprim-Sulfamethoxazole

Note: For the details on the FDA-cleared combinations of antimicrobial agents that can be tested with specific taxa, refer to the instructions for use.

Box 3. **Antimicrobial Agents Tested by the Selux AST System**

- Amikacin
- Amoxicillin-Clavulanate
- Ampicillin
- Ampicillin-Sulbactam
- Aztreonam
- Cefazolin
- Cefepime
- Cefoxitin
- Ceftazidime
- Ceftazidime-Avibactam Ceftriaxone
- Ciprofloxacin
- Eravacycline
- Ertapenem
- Gentamicin
- Imipenem-Relebactam
- Levofloxacin
- Meropenem
- Meropenem-Vaborbactam
- Minocycline
- Piperacillin-Tazobactam
- Tobramycin
- Trimethoprim-Sulfamethoxazole

Note: For the details on the FDA-cleared combinations of antimicrobial agents that can be tested with specific taxa, refer to the instructions for use.

Box 4. **Antimicrobial Agents Tested by the LifeScale Gram Negative Kit**

- Amikacin
- Ampicillin
- Aztreonam
- Cefazolin
- Cefepime
- Ceftazidime
- Ceftazidime-Avibactam
- Ertapenem
- Gentamicin
- Levofloxacin
- Meropenem
- Meropenem-Vaborbactam
- Piperacillin-Tazobactam
- Trimethoprim-Sulfamethoxazole

Note: For the details on the FDA-cleared combinations of antimicrobial agents that can be tested with specific taxa, refer to the instructions for use.

Box 5. **Antimicrobial Agents Tested by the Vitek Reveal AST System**

- Amikacin
- Amoxicillin-Clavulanate
- Ampicillin-Sulbactam
- Aztreonam
- Cefepime
- Cefotaxime
- Ceftazidime
- Ceftazidime-Avibactam
- Ceftolozane-Tazobactam
- Ceftriaxone
- Cefuroxime
- Ciprofloxacin
- Ertapenem
- Gentamicin
- Imipenem
- Levofloxacin
- Meropenem
- Meropenem-Vaborbactam
- Piperacillin-Tazobactam
- Tetracycline
- Tobramycin
- Trimethoprim-Sulfamethoxazole

Note: For the details on the FDA-cleared combinations of antimicrobial agents that can be tested with specific taxa, refer to the instructions for use.

An important component in achieving realization of the benefits of RAST will be in fostering rapid action following the results. Others have demonstrated that having a team, such as an infectious disease pharmacy team or an antimicrobial stewardship team, responsible to rapidly act on bacteremia test results can improve patient care, and integrating an antimicrobial stewardship team into the process of caring for patients with bacteremia is becoming an essential component of standard of care (10, 50, 51).

Most RAST systems require identification of the bacteria using a separate test system. It is not clear if laboratories will adopt MALDI or molecular testing methods to achieve rapid organism identification. Currently, molecular identification tests for PBC are relatively financially expensive, and MALDI workflows for rapid identification from PBC require substantial human time. However, tools are being developed to reduce the complexity of MALDI identification from PBC (bioMérieux MiTube publication pending) (52). An additional benefit of molecular tests for PBC is the detection of genes associated with resistance (e.g., *bla*_KPC_ or *mecA*), but the value of detecting these genes may decrease if RAST can be performed within a similar timeframe and with comparable clinical utility in guiding antimicrobial selection. Pairing RAST with rapid identification is essential, and the systems used for rapid identification will continue to change in parallel with RAST changes.

Many RAST systems are initially designed for PBC because of the high morbidity and mortality associated with bacteremia and the large potential to improve patient care and outcomes in patients with bacteremia. However, studies demonstrating statistically significant real-world impacts on patient outcomes attributable to RAST remain sparse. The lack of studies demonstrating an impact is likely due to the high cost required to perform these studies. The value of RAST is recognized by Medicare as it has recently awarded NTAP for use of ASTar (53), but additional studies that demonstrate a positive clinical impact of laboratory interventions would likely increase adoption of novel technologies like RAST (54).

While improving the rapidity of AST for bacteremia isolates is the application of RAST that is likely to have the largest qualitative impact on an individual patient’s outcome, improving the rapidity of UTI AST is likely to have the broadest impact on patients because of the high incidence of UTIs. However, a challenging and consequential barrier to RAST performed directly from a urine specimen is the recognition that a large minority of urine samples are contaminated with urogenital microbiota at the time of collection, and these contaminating bacteria can confound test results and interpretations. Unraveling the complexity of mixed microbiota in a primary specimen without the intermediary step of using agar culture is an important challenge. If high-quality direct-from-specimen AST is going to be able to be developed and implemented for specimens that often contain mixtures of different bacteria, such as urine or respiratory specimens, the technology needs to be able to discriminate between contaminating bacteria and bacteria causing infection. Pattern Bio is attempting to overcome this complexity using a massively parallel approach to testing individual bacteria originating from a primary specimen, and other developers may discover additional approaches that can be employed to overcome the complexity of mixed microbiota in primary specimens.

Similar to CLIA-waived molecular testing for respiratory viruses, there is market opportunity for near-patient RAST testing from urine specimens. However, beyond the technical challenges of designing an IVD that can perform accurate testing and achieve regulatory clearance, other non-technical barriers need to also be addressed. For example, increased testing costs and associated increased billing charges could be a barrier to adoption. Additionally, RAST is unlikely to be feasible for all bacterial taxa or all antimicrobials, so more traditional laboratory testing is likely to be necessary in at least a subset of circumstances. Creating workflows that enable the use of RAST systems in high-value situations while maintaining effective testing pathways for technically challenging bacteria or clinical cases that cannot be addressed using RAST systems needs to be considered.

Although PBC testing is currently a primary focus for RAST, it is possible that most AST performed in the not-too-distant future will be RAST. It is not yet clear whether this RAST will continue to be primarily performed after isolating colonies or if it might be performed directly from primary specimens containing a mixture of microbes. Additionally, work continues to be performed to attempt to use genomic data to predict antimicrobial susceptibility and resistance, which could obviate the need for a substantial portion of AST in the future (55).

## CONCLUSION

Producing timely, accurate, and actionable results is foundational to high-quality clinical microbiology practice. This minireview describes IVDs on the market or being developed that can perform RAST. These new tools can improve the timeliness of AST reporting, which has the potential to improve the quality of patient care that can be offered by clinical microbiology. The clinical microbiology community will need to assess the accuracy and clinical impact of these test systems in the years ahead.
